# Mitotic activity index and CD25+ lymphocytes predict risk of stage progression in non-muscle invasive bladder cancer

**DOI:** 10.1371/journal.pone.0233676

**Published:** 2020-06-02

**Authors:** Melinda Lillesand, Vebjørn Kvikstad, Ok Målfrid Mangrud, Einar Gudlaugsson, Bianca van Diermen-Hidle, Ivar Skaland, Jan P. A. Baak, Emiel A. M. Janssen

**Affiliations:** 1 Department of Pathology, Stavanger University Hospital, Stavanger, Norway; 2 Department of Mathematics and Natural Science, University of Stavanger, Stavanger, Norway; 3 Department of Pathology, Innlandet Hospital, Lillehammer, Norway; 4 Jan Baak AS, Tananger, Norway; Chang Gung Memorial Hospital at Linkou, TAIWAN

## Abstract

In urothelial cell type non-muscle invasive urinary bladder carcinoma, TNM stage and WHO grade are widely used to classify patients into low and high‑risk groups for prognostic and therapeutic decision-making. However, stage and grade reproducibility and prediction accuracy are wanting. This may lead to suboptimal treatment. We evaluated whether proliferation features, nuclear area of the epithelial cancer cells and the composition of stromal and tumor infiltrating lymphocytes have independent prognostic value. In 183 primary non-muscle invasive bladder cancer patients with long follow-up (median for stage progression cohort: 119 months, range 5-173; median for tumor recurrence cohort: 82, range 3-165) proliferation features Ki67, PPH3 and Mitotic Activity Index (MAI), Mean Nuclear Area (MNA), lymphocyte subsets (CD8+, CD4+, CD25+) and plasma cells (CD138+) were assessed on consecutive sections. Post-resection instillation treatments (none, mitomycin, BCG) were strictly standardized during the intake period. Risk of recurrence was associated with expression of Ki67 (≤ 39 vs. > 39) and Multifocality *(p* = 0.01). Patients with low Ki67 had a higher recurrence rate than those with high Ki67. Lymphocyte composition did not predict recurrence. Stage progression was strongly associated with high values for MAI (>15) and CD25+ (>0.2%). In a multivariate analysis the combination of MAI and CD25+ was the single most prognostic feature (*p*<0.001). Validation of these results in additional, independent studies is warranted.

## Introduction

Urothelial cell carcinoma (UCC) is the most common type of carcinoma of the urinary bladder, accounting for about 90% of cases in Western Europe [1]. About 75 to 80% of the UCC are non-muscle invasive bladder cancer (NMIBC) at the time of diagnosis [2] and approximately 5 to 10% of these progress to muscle invasive disease [3, 4]. In addition to tumor stage (TNM-classification), grading based on the degree of anaplasia is used as an important prognostic factor. A recent review reported that WHO1973 and WHO04/WHO16 grading systems are suboptimal concerning both reproducibility and prognostic value [5, 6]. On the other hand, earlier reports stated that proliferation features Ki67, PPH3 and Mitotic Activity Index (MAI), as well the nuclear feature Mean Nuclear Area of the largest 10 nuclei (MNA), were strongly predictive, prognostic and cost effective markers, overriding both TNM stage and grade in NMIBC [7–9]. Currently, intravesical immunotherapy (BCG) is the standard treatment for intermediate or high-risk patients according to European Association of Urology guidelines [10]. Due to the intensive follow-up and treatment side effects, the costs per bladder cancer patient are high compared to other cancer types [11]. As such better predictive and prognostic markers than stage and grade are warranted [12].

Several publications have shown that an increased level of tumor infiltrating lymphocytes (TILs) is associated with a better overall survival [13] in patients with urothelial carcinomas [14]. More specifically, CD8+ cytotoxic T cells were related to a more favorable clinical outcome in both invasive bladder cancer [15] and many other tumor types. Also, the ratio of CD4+ and CD8+ TILs showed an altered pattern in recurrent and non-recurrent tumors in patients with NMIBC [16]. In addition, a strong association between increased numbers of regulatory T cells (Tregs) and bladder tumor recurrence, metastasis and stage has been reported [17]. Similarly, the ratio between tumor infiltrating effector T cells and Tregs was inversely related with tumor recurrence in invasive urothelial carcinomas [18].

Unfortunately, most of these studies have included small numbers of patients and mixed NMIBC and higher stage muscle invasive bladder cancer (MIBC) [16]. Furthermore, follow-up was often short, in spite of the fact that recurrences can also occur after many years. Another issue is that the selection of positive and negative cells were not random in the measurement procedure. This may have caused serious selection bias and erroneous results. At present, there are no reliable data regarding the significance of stromal and tumor infiltrating lymphocytes on prognosis in pTa pT1 urothelial carcinomas.

The aim of the present study is to investigate, whether CD8+, CD4+, CD25+ lymphocytes, and CD138+ plasma cells (immune cell markers) have additional prognostic value for recurrence and stage progression in a homogeneous cohort of pTa-pT1 tumors with long follow-up. We followed a fully randomized selection procedure for the measurement of Ki67, MNA and immune cell markers within the least differentiated area of the tumors. We hypothesize that adaptive immune cell composition in addition to proliferation features and MNA can have an additional value to predict, recurrence and stage progression.

## Material and methods

This study was approved by the Norwegian Regional Ethics Committee (REK Vest, #106/09) before the start of the study. With approval from REK Vest, informed consent was not obtained as the tissue samples had already been removed for diagnostic and treatment purposes. In the period between January 1, 2002 and December 31, 2007, 249 patients were diagnosed with primary NMIBC, at the Department of Pathology, Stavanger University Hospital (SUH). Sixty-six cases were lost to follow-up or had inadequate sample quality for further analysis, leaving 183 patients to be included in the study (Table 1). Tissue samples were obtained by TURB or biopsies from the urinary bladder mucosa. After TURB, most patients underwent a single installation of the cytotoxic agent mitomycin C, while primary, high-risk patients (13%) classified according to national guidelines (bladdercalculator.no) were treated with BCG immunotherapy. All specimens were staged and graded by four experienced pathologists (VK, OM, EG and JPAB) according to the WHO73 and WHO04 grading systems [19]. Tumor recurrence was defined as the presence of (a) tumor(s) in the bladder mucosa more than 3 months after the primary diagnosis. Total follow-up time, registered for statistical analyses of recurrence, was defined as the time from primary diagnosis until last control with cystoscopy. Stage progression was defined as recurrent tumor with pT2 or higher stage or confirmed metastasis, more than 3 months after primary diagnosis. Follow-up time registered for statistical analyses of stage progression was from primary diagnosis until death, or last known contact with the health care system. For stage progression, clinical follow-up was regarded as enough, but for recurrence, regular scheduled follow-up with cystoscopies according to guidelines was considered necessary. For the calculation of tumor recurrence only 177 patients were included as 6 more patients were lost to follow-up, as patients had to keep their urinary bladders and go through cystoscopy at least 3 months after primary diagnosis. Follow‑up data were retrieved from medical hospital records, with last registration on June 30, 2016.

**Table 1:** Exclusion criteria, number of excluded and included patients.

Primary pTaT1 urothelial carcinomas at SUH 2002–2006	249
Insufficient material	21
Thermal damage	11
Fragmented specimen	1
Necrotic specimen	2
Sarcomatoid differentiation	1
Previous urothelial carcinoma (on review of clinical notes)	1
cT3 or cT4 (on review of clinical notes)	3
pT2 at re-TURB	2
pT2 at review	1
Clinical metastasis at time of diagnosis	2
Lost to follow-up	11
Insufficient material for quantification of immune cell markers	2
Metastases at renal pelvis, ureter and urethra	8
Included in study	**183**

### Immunohistochemistry (IHC)

TURB and biopsies were fixed in 10% neutral buffered Formalin®, dehydrated, and embedded in paraffin. Sections for assessment of MAI, MNA and histology were stained by Hematoxylin, Erythrosine & Saffron (HES). Adjacent to the HES stained sections, consecutive 4 μm paraffin sections were mounted onto Superfrost Plus® slides (Menzel, Braunschweig, Germany) and dried overnight at 37°C followed by 1 h at 60°C. Deparaffinization was performed stepwise by xylene, thereafter rehydration through decreasing concentrations of alcohol solutions. Heat-mediated antigen retrieval was performed with a computerized retrieval system (Immuno-Prep©; Instrumec, Oslo, Norway) using TRIS (10 mM) - EDTA (1 mM) antigen retrieval buffer (pH 9). The deparaffinized sections were first heated for 3 min at 110°C and thereafter incubated for 10 min at 95°C and finally cooled to 20°C [20], in a pressure cooker. For the elimination of nonspecific background, a Tris-Buffered Saline Solution (DAKO, Glostrup, Denmark, S1968), containing 0.05% Tween 20, was used as a wash buffer (pH 7.6). Endogenous peroxidase activity was inactivated by the incubation of tissue sections in the peroxidase-blocking reagent (DAKO, Glostrup, Denmark; S2001) for 10 min. Immunostaining of CD4+, CD8+, CD25+ T lymphocyte subsets, CD138+ plasma cells and proliferation marker Ki67 was performed using an Autostainer (DAKO, Glostrup, Denmark). The tissue sections were incubated with the monoclonal antibodies using the following dilutions: CD4 (Novocastra, Newcastle upon Tyne, UK; clone 1F6, 1:20); CD8 (DAKO, Glostrup, Denmark; clone C8/144B, 1:50); CD25 (Novocastra, Newcastle upon Tyne, UK; clone 4C9, 1:150); CD138 (Serotec, Kidlington, UK; clone B‑B4, 1:200) and Ki67 (DAKO, Glostrup, Denmark; clone MIB-1, 1:100). An antibody diluent (DAKO, Glostrup, Denmark; S0809) was used for the preparation of primary and secondary antibody dilutions. The immune complex was visualized by peroxidase/DAB (DAKO, Glostrup, Denmark; EnVision Detection System, K5007) with incubation of Envision/HRP, rabbit/mouse antibody (ENV) for 30 min and DAB-chromogen with hematoxylin counterstain for 10 min. Thereafter the tissue sections were dehydrated and mounted [7, 21].

### Quantitative image analysis

**Fig 1:**
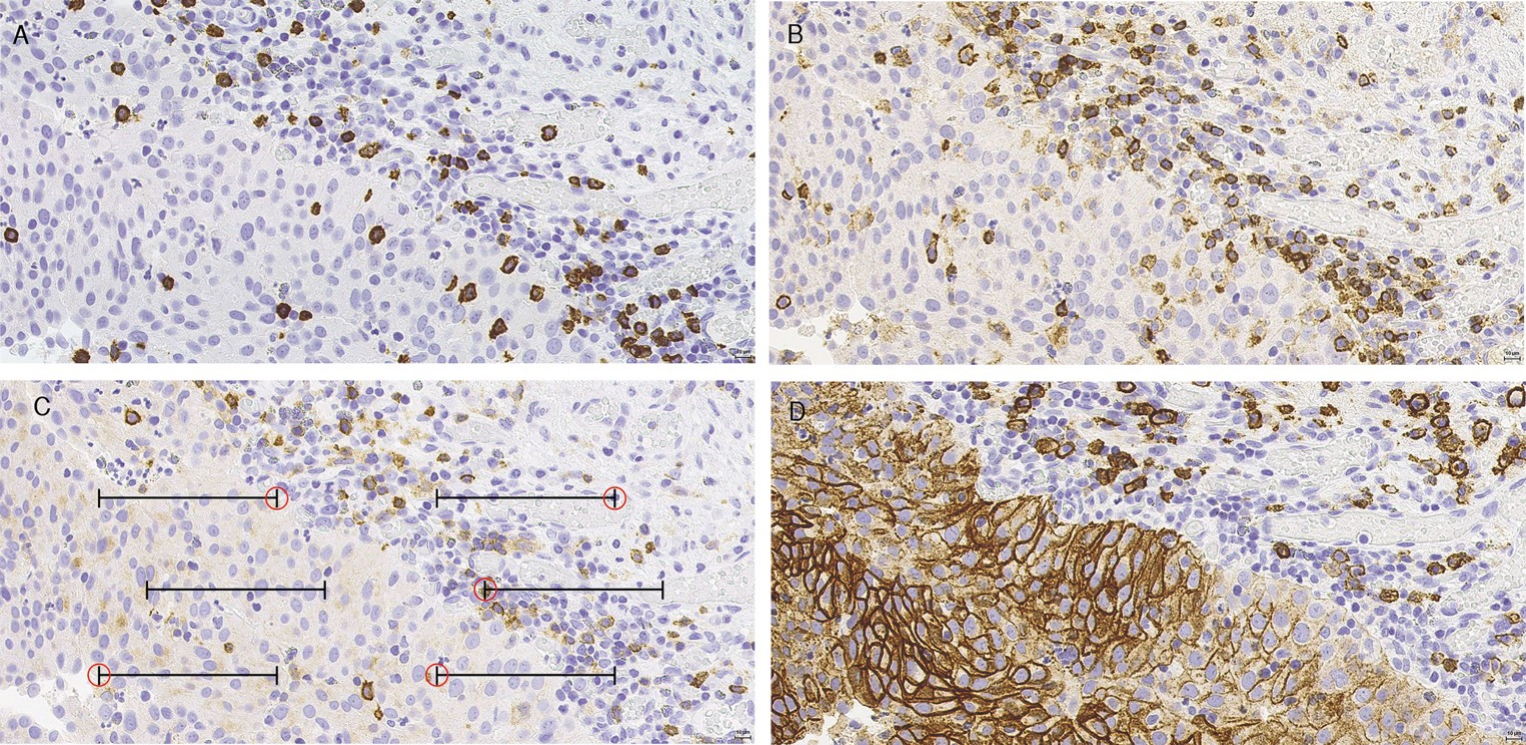
Immune cell markers CD8, CD4, CD25 and CD138 in representative urothelial bladder cancer tissue. (A) CD8 IHC stain (400X magnifications), (B) CD4 IHC stain (400X magnifications), (C) CD25 IHC stain (400X magnifications) (D) CD138 IHC stain (400X magnifications). In each field of vision positively stained immune cells were quantified as “positive counts” and negatively stained immune cells and other cell types were quantified as “negative counts”. Counts were registered using six electronic grids with five endpoints. Scale bar 10 μm.

In each case the least differentiated area was carefully selected and demarcated on HES stained sections by a pathologist, based on the degree of cellular anaplasia. All assessments were done in this demarcated area. MAI, PPH3 and MNA were assessed as previously described [7–9]. Highly reproducible, semi-automated quantification of all immune cell markers and Ki67 was performed by using the motorized semi-automated QPRODIT (version 6.1) interactive image analysis system (Leica, Cambridge, UK). Immune cell quantification was performed at a final magnification of 400X using a 6-line grid in 150 random fields of vision (FOV) within the measurement area. In each FOV, the same endpoints of six electronic gridlines were used to register both immunohistochemically stained (IHC) positive-, IHC-negative immune cells and other cell types. Consequently, the sampling procedure within the measurement area was fully at random, which is essential for getting a well reproducible and prognostically accurate results (see Fig 1). Quantification and percentage calculation of Ki67-positivity was performed as earlier described [7–9]. The percentage of immune cell marker positivity was defined as: ((IHC positive immune cells) / (Total numbers of cells within the measurement area)) x 100. The average total counted cells within the measurement area was 210 cells (range 30-396). The average measurement area was 15 mm^2^ (range 2-91).

### Statistics

Statistical analysis was performed by using SPSS, version 21 (SPSS Inc., Chicago, IL, USA) and MedCalc Statistical Software version 19.1 (MedCalc Software BV, Ostend, Belgium; https://www.medcalc.org; 2019). The immunological markers, proliferation features and MNA were continuous variables, whilst stage progression, recurrence, grade and stage were categorical variables. Different percentiles (medians, tertiles and quartiles) and ROC curve analyses were used to determine the optimal prognostic thresholds of the continuous variables. Proliferation features and MNA were dichotomized by using previously published prognostic thresholds in both recurrence and stage progression cohorts [7–9]. In addition, proliferation features and MNA were dichotomized by using median values as well in the recurrence cohort. Univariate, nonparametric Kruskal-Wallis and Mann Whitney-U tests were performed to compare differences of continuous variables in the independent groups. A log rank test was run to determine if there were differences in the survival distributions of recurrence or stage progression for the two subgroups of immune cell markers, proliferation features and MNA or clinical and histopathological parameters. The differences between the subgroups were considered significant if the probability of no difference (*p* value) was <0.05. Univariate Cox proportional hazard ratios (HR) with 95% confidence intervals (CI) were also calculated. Multivariate Cox survival analysis was performed to evaluate the best prognostic combination of both continuous and categorical variables.

## Results

The median follow-up time of the 177 patients available for recurrence analysis, was 82 months (range 3 to 165). From these, 105 patients (60%) experienced tumor recurrence. When analyzing for stage progression, the median follow-up time was 119 months (range 5 to 173). From these 183 patients, 13 patients (7%) experienced stage progression. In both groups, the gender distribution of the patients was 76% men and 24% women, and median age at first diagnosis was 74 years (range 39 to 95). According to the TNM-classification in both groups, 80% of the tumors presented as stage pTa and 61% were classified as WHO04 low-grade urothelial carcinoma. The distribution of WHO73 classification G1, G2 and G3 was 23%, 51% and 26% respectively.

### Recurrence analysis

**Fig 2:**
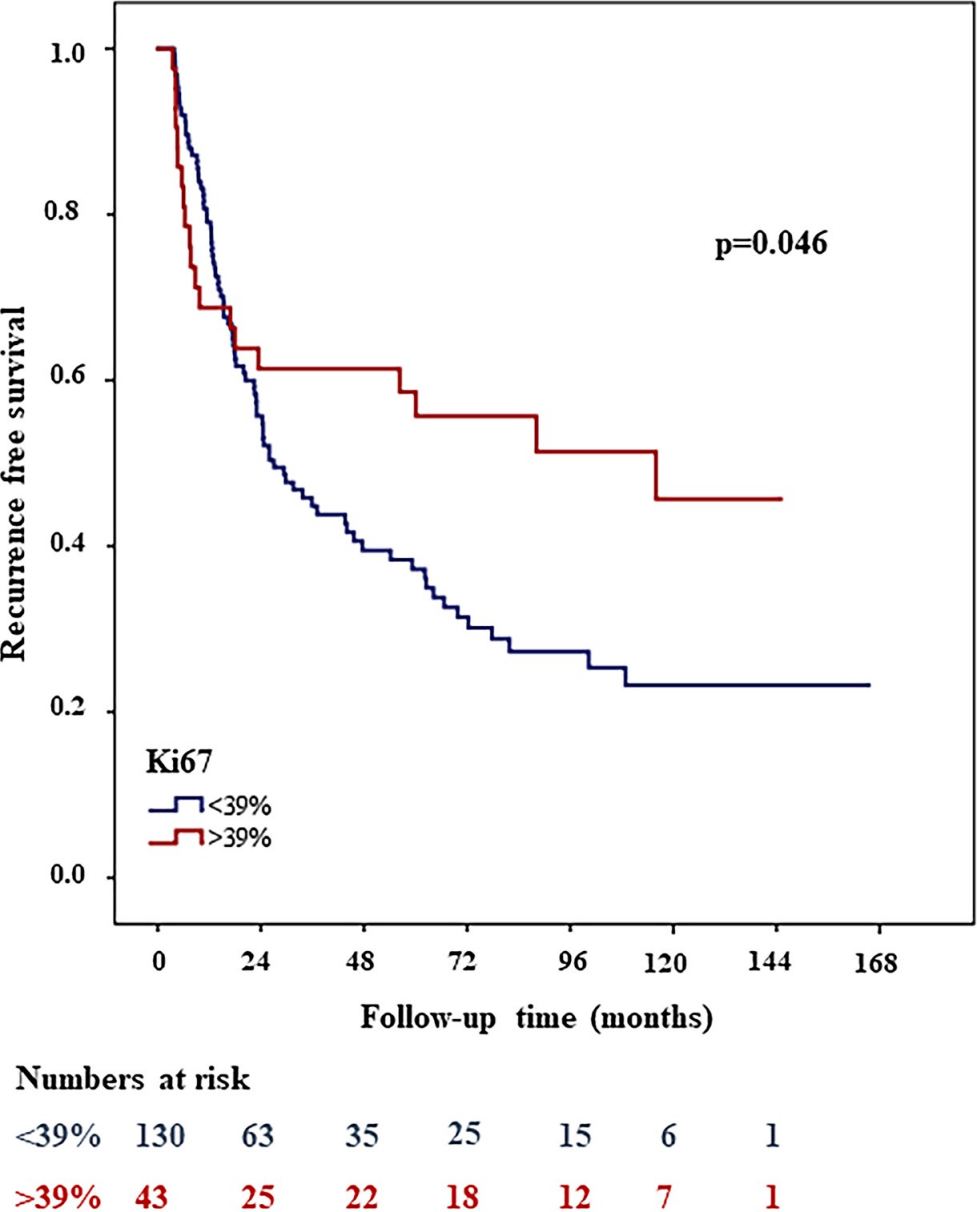
Low Ki67 (≤39%) associated with shorter recurrence free survival in Kaplan Meier survival analysis.

**Fig 3:**
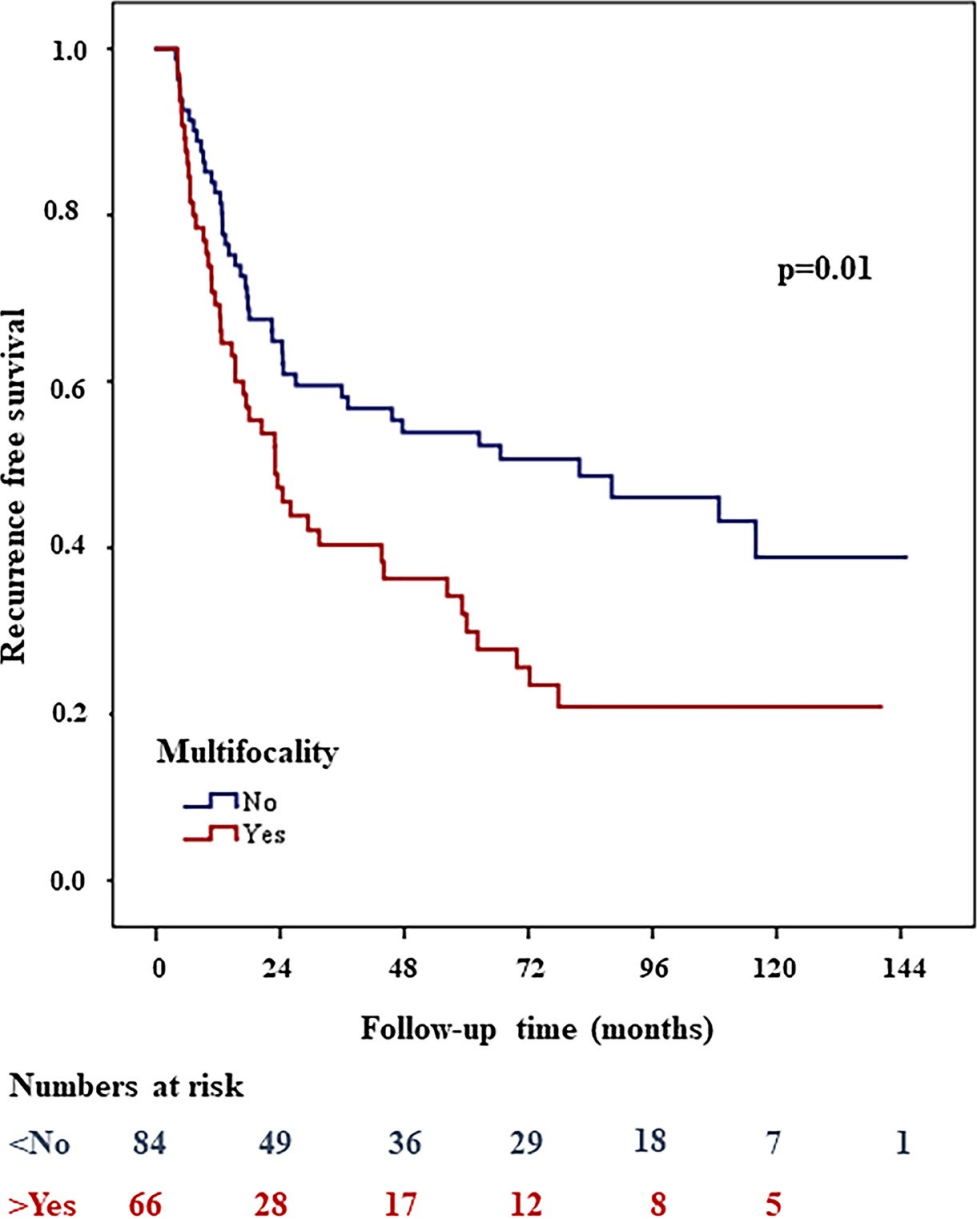
High CD25+ (>0.2%) associated with shorter stage progression free survival in Kaplan Meier survival analysis.

In total 173/177 and 150/177 patients were available for statistical analyses of Ki67 and Multifocality respectively. Out of all investigated immune cell markers, proliferation and nuclear features, clinical and histopathological parameters only Ki67 (threshold 39%, HR: 0.61, 95% CI, 0.4-0.9; *p* = 0.05) and Multifocality (HR: 1.8, 95% CI, 1.2-2.7; *p =* 0.01) showed significant association with tumor recurrence. Fig 2 shows the Kaplan-Meier curves for recurrence free survival for the two subgroups of Ki67. The group with low Ki67 had significantly shorter recurrence free survival, than the group with higher values. The presence of Multifocality correlated with a shorter recurrence free survival (Fig 3). There were no statistically significant differences between the median values of the immune cell markers in patients with or without recurrence. Median values and range as well threshold values; and hazard ratio, CIs, and *p* values for histopathological characteristics, proliferation features, MNA and immune cell markers were calculated by univariate recurrence free survival analyses summarized in Table 2.

**Table 2:** Univariate analyses for recurrence free survival of histopathological characteristics, immune cell markers, proliferation features and MNA.

Characteristics	Tumor recurrence cohort (105/177)			
	Event/at risk (%)	Log rank P value	HR	95% CI
Age at diagnosis				
<74	51/87 (59)	0.12	1.4	0.9-2.0
≥74	54/90 (60)			
(range 39-95)				
Gender				
Male	77/135 (57)	0.73	1.1	0.7-1.7
Female	28/42 (66)			
WHO1973 grade				
1	27/41 (66)	0.39		
2	50/90 (56)		0.8	0.5-1.2
3	28/46 (61)		1.0	0.6-1.7
WHO2004 grade				
Low	67/108 (62)	0.92	1.0	0.7-1.5
High	38/69 (55)			
Stage				
Ta	85/142 (60)	0.63	1.1	0.7-1.8
T1	20/35 (57)			
Multifocality				
No	42/84 (50)	**0.01**	1.8	1.2-2.7
Yes	47/66 (71)			
CIS				
No	92/156 (59)	0.63	1.2	0.6-2.1
Yes	13/21 (62)			
CD25+ (%)				
≤0.2	52/89 (58)	0.51	1.1	0.8-1.7
>0.2	53/88(60)			
(range 0-10)				
CD8+ (%)				
<3.0	53/89 (60)	0.74	1.1	0.7-1.6
≥3.0	52/88 (59)			
(range 0-28)				
CD4+ (%)				
<4.5	55/89 (62)	0.83	1.0	0.7-1.4
≥4.5	50/88 (57)			
(range 0–57)				
CD138+ (%)				
<1.4	53/89 (60)	0.62	1.1	0.8-1.6
≥1.4	52/88 (59)			
(range 0-20)				
Ki67 (%)				
≤39	83/130 (64)	**0.05**	0.6	0.4-0.9
>39	20/43 (47)			
(range 1-82)				
Ki67 (median)				
<18	53/86 (62)	0.95	1.0	0.7-1.5
≥18	50/87 (57)			
(range 1-82)				
PPH3				
<40	79/131 (60)	0.52	0.86	0.5-1.4
≥40	25/44 (57)			
(range 0-137)				
PPH3 (median)				
<17	53/84 (63)	0.93	1.0	0.7-1.5
≥17	51/91 (56)			
(range 0-137)				
MAI				
≤15	80/138 (58)	0.56	1.1	0.7-1.8
>15	25/39 (64)			
(range 0-46)				
MAI (median)				
<4	51/83 (61)	0.86	1.0	0.7-1.5
≥4	54/94 (57)			
(range 0-46)				
MNA (mm^2^)				
≤170	90/154 (58)	0.26	1.4	0.8-2.4
>170	15/23 (65)			
(range 49-488)				
MAI CD25+ (%)				
Low-Low*	45/77 (58)	0.6		
Mixed*	42/73 (57)		1.0	0.7-1.6
High-High*	18/27 (67)		1.3	0.8-2.3

*HR* Hazard Ratio, *CI* Confidence interval

*Low-Low: low CD25 low MAI, *Mixed*: low CD25 high MAI and high CD25 low MAI, *High-High*: high CD25 high MAI

### Stage progression analysis

**Fig 4:**
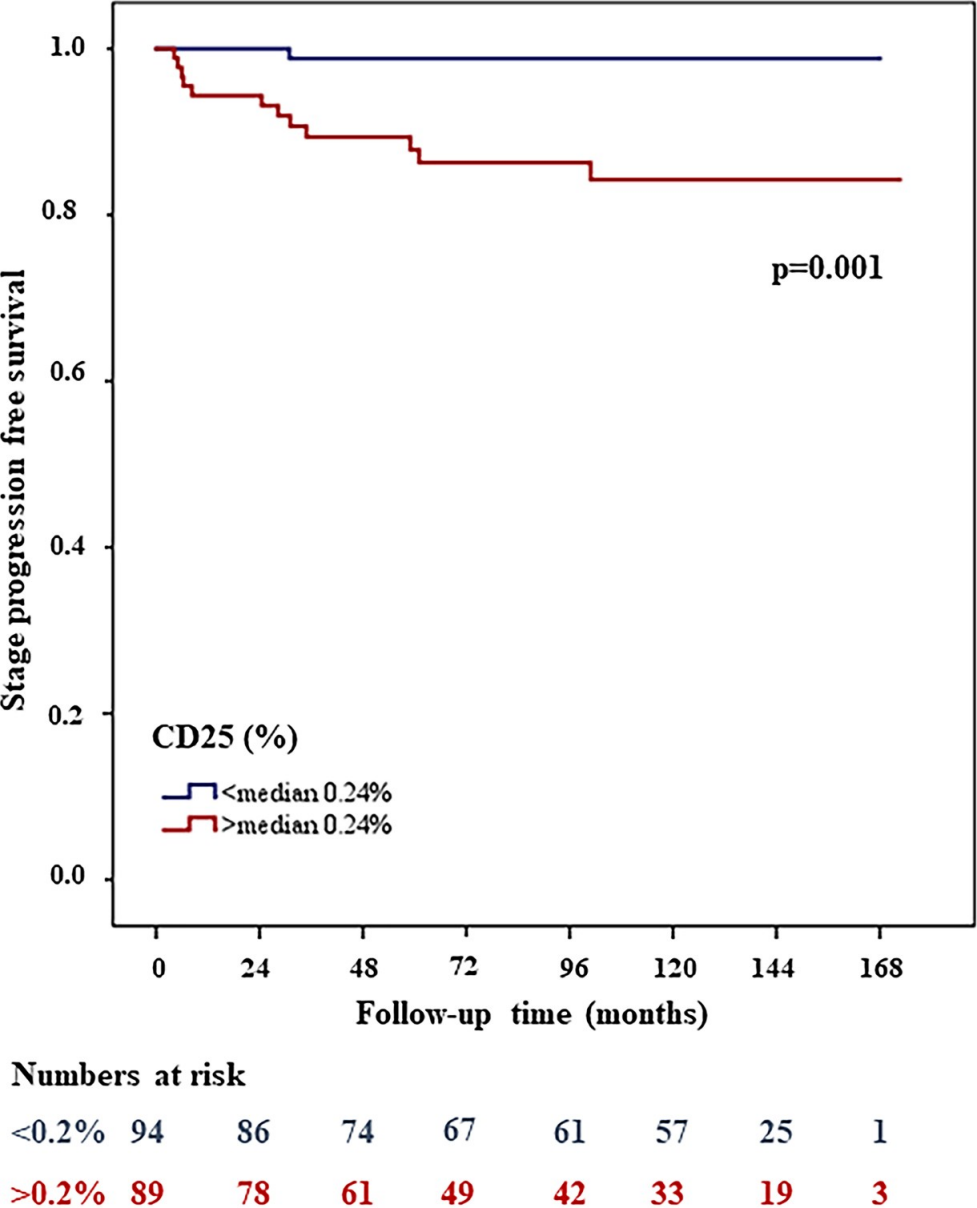
High MAI (>15) associated with shorter stage progression free survival in Kaplan Meier survival analysis.

In our stage progression cohort, 183 NMIBC patients were available, 146 with pTa tumors and 37 with pT1 tumors. Interestingly, in pT1 tumors the percentages of CD25+, CD4+ and CD138+ cells were significantly higher than in pTa tumors (*p*<0.001, *p* = 0.01 and *p* = 0.01 respectively). When examining all 183 pTaT1 patients, out of all immune cell subsets only CD25+ showed significant association with stage progression (HR: 13.8, 95% CI, 1.8-106.2; *p* = 0.001) (Fig 4). Median values and range as well as threshold values; and hazard ratio, CIs, and *p* values for histopathological characteristics, proliferation features, MNA and immune cell markers were calculated by univariate recurrence free survival analyses summarized in Table 3. Patients with higher stage, grades and concomitant carcinoma in situ (CIS) had significantly higher stage progression risks (*p*<0.05).

**Table 3:** Univariate analyses for stage progression free survival of clinical and histopathological characteristics, immune cell markers, proliferation features and MNA.

Characteristics		Stage Progression cohort (13/183)			
		Event/at risk (%)	Log rank P value	HR	95% CI
Age at diagnosis					
<74		3/90 (3)	**0.02**	4.0	1.1-14.7
≥74		10/93(11)			
(range 39–95)					
Gender					
Male		11/140 (8)	0.50	0.6	0.1-2.7
Female		2/43 (5)			
WHO1973 grade					
1		1/41 (2)	**0.04**		
2		5/94 (5)		2.3	0.3-19.3
3		7/48 (15)		6.8	0.8-55.6
WHO2004 grade					
Low		4/113 (3)	**0.01**	4.1	1.3-13.2
High		9/70 (13)			
Stage					
Ta		4/146 (3)	**<0.001**	10.8	3.3-35.3
T1		9/37 (24)			
Multifocality					
No		3/87 (3)	0.10	3.0	0.8-11.5
Yes		7/68 (10)			
CIS					
No		9/162 (6)	**0.01**	4.3	1.3-14.0
Yes		4/21 (19)			
CD25+ (%)					
≤0.2		1/94 (1)	**0.001**	13.8	1.8-106.2
>0.2		12/89 (13)			
(range 0–10)					
CD8+ (%)					
<3.0		7/92 (8)	0.87	0.9	0.3-2.7
≥3.0		6/91 (7)			
(range 0–28)					
CD4+ (%)					
<4.5		5/92 (5)	0.31	1.8	0.6-5.4
≥4.5		8/91 (9)			
(range 0–57)					
CD138+ (%)					
<1.4		5/92 (5)	0.33	1.7	0.6-5.3
≥1.4		8/91 (9)			
(range 0–20)					
Ki67 (%)					
≤39		7/135 (5)	0.12	2.4	0.8-7.6
>39		5/44 (11)			
(range 1–82)					
Ki67 (median)					
<18		1/89 (1)	**0.003**	11.5	1.5-88.9
≥18		11/90 (12)			
(range 1–82)					
PPH3					
<40		4/136 (3)	**<0.001**	7.3	2.3-23.8
≥40		9/45 (20)			
(range 0–137)					
MAI					
≤15		5/143 (3)	**<0.001**	6.8	2.2-20.7
>15		8/40 (20)			
(range 0–46)					
MNA (mm2)					
≤170		10/159 (6)	**0.20**	2.3	0.6-8.2
>170		3/24 (12)			
(range 49–488)					
MAI CD25+ (%)					
Low-Low*		0/81 (0)	**<0.001**		
Mixed*		6/75 (8)		-	-
High-High*		7/27 (26)		-	-

*HR* Hazard Ratio, *CI* Confidence interval

*Low-Low: low CD25 low MAI, *Mixed*: low CD25 high MAI and high CD25 low MAI, *High-High*: high CD25 high MAI

**Fig 5:**
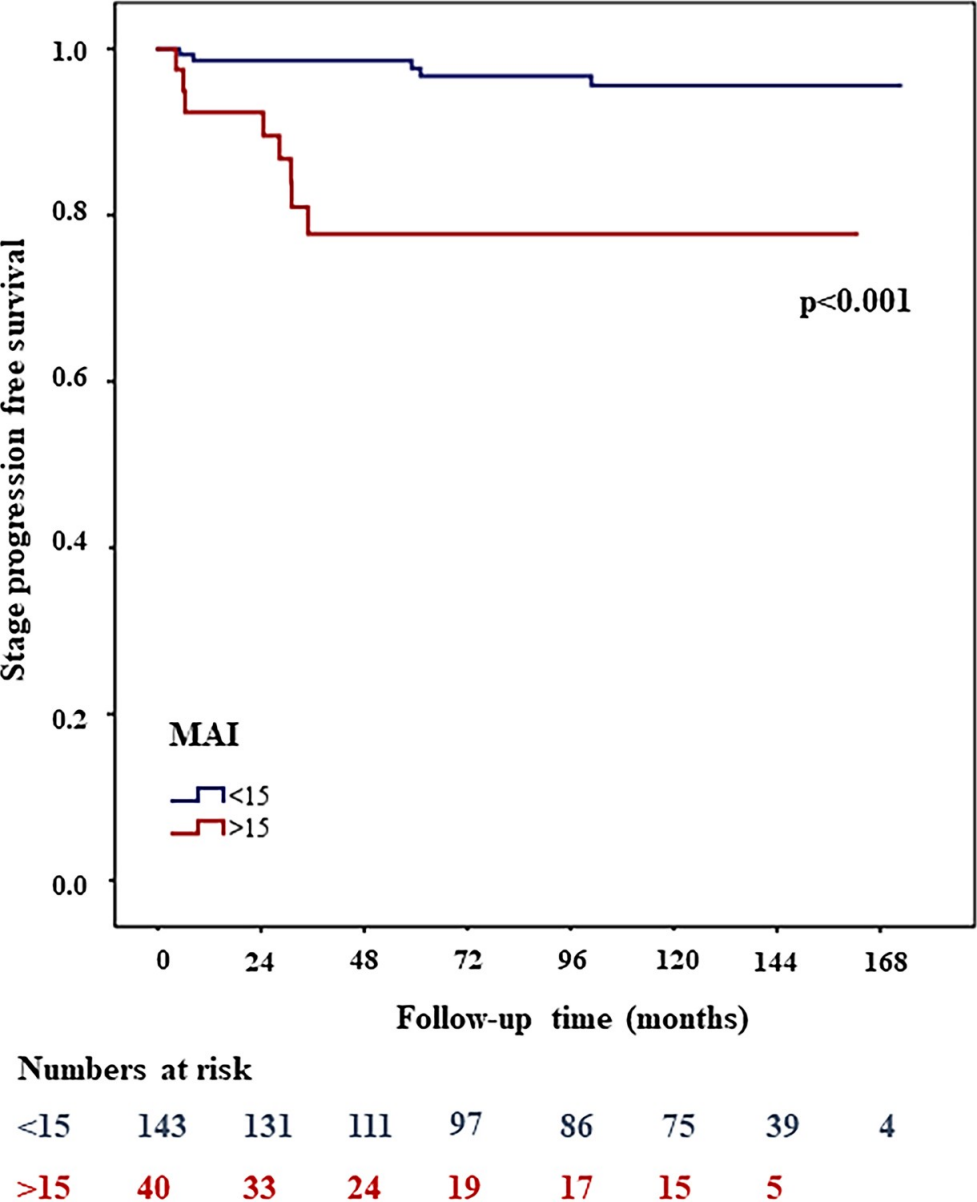
The combination of MAI and CD25+ stratifies patients into three groups. Patients with both low MAI and CD25+ values showed a better outcome than those with high amount of MAI and/or CD25+.

**Fig 6:**
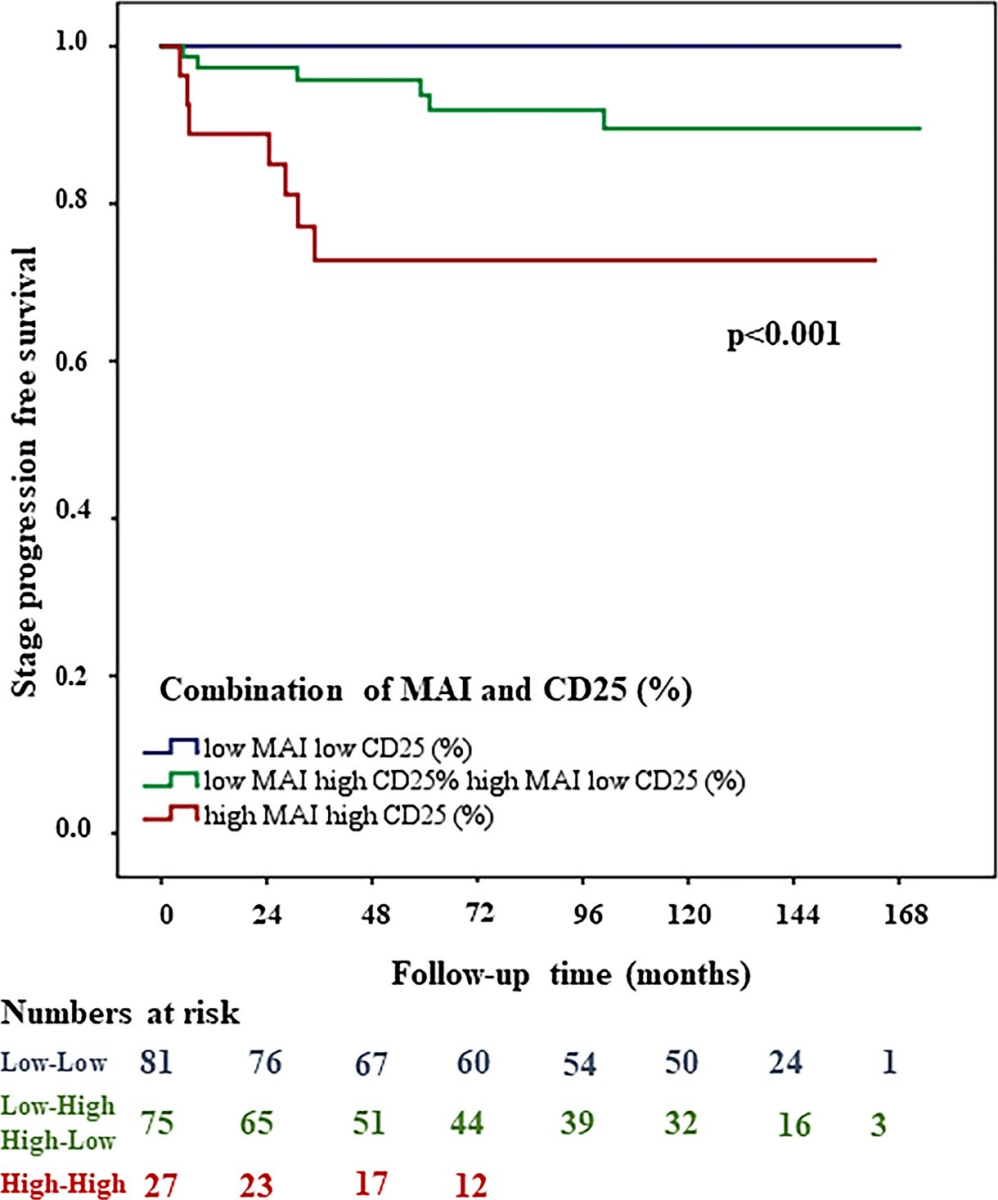


Multivariate Cox proportional hazard analysis of all 183 patients, including age, WHO1973 and WHO2004 grade, KI67 (threshold≥18), PPH3, MAI and CD25+, showed that MAI (threshold >15) was the strongest single predictor for stage progression (HR: 8.6, 95% CI, 2.6-28.5; *p<*0.001). When the combination of MAI and CD25+ was also included, the MAI CD25+ combination was an even better predictor for stage progression (HR, 95% CI could not be computed, *p<*0.001). With Kaplan-Meier survival analyses, 26% of patients experienced stage progression in the high MAI high CD25+ group and 0% experienced stage progression in the low MAI low CD25+ group. In the mixed group (low MAI high CD25+, high MAI low CD25+) 8% of patients progressed. Fig 5 shows the stage progression free survival curves for the two subgroups of MAI and Fig 6 shows the progression free survival curves for the two subgroups of MAI CD25+ combination.

## Discussion

In the last decades, several studies have analyzed the association between different subgroups of lymphocytes and clinical outcome in bladder cancer. However, both concordant and conflicting results are observed when comparing our findings. One explanation might be the variation between the investigated areas within the bladder mucosa. Alternatively, numerous subsets of CD25+, CD4+ and CD8+ lymphocytes may coexist in the tumor microenvironment, which could differ in phenotypes, functions and locations in different time perspectives. One study demonstrated that increased numbers of CD4+ predict a lower 5-year overall survival (OS) in NMIBC [22]. On the other hand, it was also reported that increased numbers of CD4+ cells are related to a prolonged recurrence free survival in high-risk NMBIC [23]. Others published that CD8+ TILs are associated with better disease-free and overall survival in more advanced tumors [15, 24]. However Zhang *et al*. demonstrated that higher numbers of CD8+ TILs were related to a more unfavorable clinical outcome in pTa-pT2 (organ confined) tumors [25]. Regarding Tregs, one study reported that high FOXP3+/CD3+ and FOXP3+/CD8+ cell ratios predict poorer overall survival in pT1-pT4 tumors [26]. Controversially, another study, observing pT1-pT4 tumors as well, demonstrated that high numbers of FOXP3+ lymphocytes were correlated with better survival [27].

An important issue when comparing these studies is the fact that many have included patients ranging from pTa-pT4. Furthermore, different methods and measurement areas have been used (some only intrastromal, others within the tumor urothelium as well) to quantitate the different subsets of lymphocytes. Moreover, non-random selection methods for the measurements have been described, which can be a serious cause for biased results. In the present study we have prevented these methodological challenges (consecutive sections, only pTa pT1 tumors, random selection methods for quantification) and analyzed the association between CD8+, CD4+, CD25+, CD138+, proliferation and nuclear features, histopathological and clinical parameters and outcomes (recurrence and stage progression free survival).

Doing so, the results for recurrence were different from those before. Low percentage of Ki67 was associated with a high risk for tumor recurrence. These results are unexpected and contrary to a recent meta-analysis, which showed that in 34 studies, high Ki67 was related to poor recurrence free survival [28]. One of the explanation could be, that in this meta-analysis Ki67 threshold varied between 5% and 25%, in our study it was much higher, 39% (previously published prognostic threshold was used) [7]. In addition Ki67 positive cell quantification procedures differed from our highly reproducible semi-automated quantification method [7–9]. On the other hand the previously described threshold was calculated in correlation to progression, another threshold might be necessary for the prediction of recurrence. When using the median value for any of the proliferation markers no significant correlation was found with recurrence (Table 2). As such we need to validate the prognostic value of Ki67 in larger, independent, cohorts.

In our investigation we were not able to confirm a connection between the amount of CD8+, CD4+ and CD138+ cells, tumor recurrence or stage progression, nor the association between the amount of CD25+ cells and tumor recurrence in patients with NMIBC as described by other authors. Although our findings on CD4+ cells, are similar to the results of Kripna *et al*., who also reported no significant difference in the number of CD4+ cells between the recurrent and non-recurrent group in low-grade papillary urothelial carcinoma. However, they found an association between an increased number of CD8+ TILs and tumor recurrence [16]. Furthermore, Pichler *et al*. demonstrated that high FOXP3+CD25+ Tregs was associated with shorter recurrence free survival in NMIBC, which we could not confirm in our recurrence cohort [23].

As to stage progression prediction, we show that the number of CD25+ cells differed strongly between pTa and pT1, and is also significantly associated with stage progression. Loskog *et al*. previously published a similar finding that tumor infiltrating CD4+CD25+ T cells show a regulatory phenotype in human bladder cancer biopsies which was strongly associated with tumor progression [29]. Our results as well suggest a systemic suppression of immune response by CD4+CD25+ Tregs in the bladder tumor tissue. Furthermore, the combination of MAI and CD25+ was the strongest predictor for tumor stage progression and strongly associated to TNM stages. The combination of MAI and CD25+ could define patient groups even better and in a more standardized and reproducible manner and can identify a large group of patients with a (nearly) 100% stage progression-free survival. Based on our current data and previously published data, we hypothesize that the CD25+ cells are Tregs, which increase in numbers following the development from superficial to more advanced stages, and as such tumors develop a gradually more immunosuppressive and more heterogeneous/proliferative tumor microenvironment.

A weakness of our study is, that in spite of the large number of patients and long follow-up, the number of patients with stage progression is still limited (n = 13). Mangrud *et al*. published, that the threshold estimation of Ki67 and other proliferation markers (MAI and PPH3) was sensitive to the number of patients [7]. Therefore external validation of our results is essential. Another issue is, that threshold values for Ki67 differed between previously published cohorts as well [28]. One of the explanations could be the lack of standardization of Ki67 antibodies, which makes the interpretation of true positive and negative cells difficult. In addition we used the same threshold values in both recurrence and stage progression cohorts. On the other hand Kaplan Meier survival plots and ROC curve analyses could not estimate an optimal threshold value in the recurrence cohort. Furthermore, tumor size of the patients (>3 cm) were not available in our retrospective cohort, which is an important factor regarding tumor recurrence. Although our quantification method for the immunohistochemical markers is highly reproducible, the method is very labor intensive. Therefore independent studies are needed to validate our results using more sophisticated and fully automated digital image analyses such as artificial intelligence. Bunimovich-Mendrazitsky recently published a mathematical dynamic model as a powerful tool, which could be used to analyze the interactions between stromal and tumor infiltrating lymphocytes and tumor cells [30] and to develop a standardized immunoscore [31] for predicting clinical outcome of patients with NMIBC.

Overall, the findings of our study show that a combination of MAI and CD25+ have overridingly strong prognostic value to predict stage progression and are worth validating in a well-defined, larger cohort.
